# Henoch–schonlein purpura following exposure to SARS-CoV2 vaccine or infection: a systematic review and a case report

**DOI:** 10.1007/s11739-023-03366-w

**Published:** 2023-07-27

**Authors:** Federica Di Vincenzo, Sara Ennas, Marco Pizzoferrato, Stefano Bibbò, Serena Porcari, Gianluca Ianiro, Giovanni Cammarota

**Affiliations:** 1 UOC di Gastroenterologia, Dipartimento di Scienze Mediche e Chirurgiche, Fondazione Policlinico Universitario A. Gemelli IRCCS, L. go A. Gemelli 8, Roma, Italia; 2 Dipartimento di Medicina e Chirurgia Traslazionale, Università Cattolica del Sacro Cuore, L. go F. Vito 1, Roma, Italia

**Keywords:** Henoch–Schonlein purpura, IgA-mediated vasculitis, SARS-CoV2 vaccination, SARS-CoV2 infection

## Abstract

**Background:**

Henoch–Schonlein purpura (HSP) is an IgA-mediated systemic small-vessel vasculitis (IgAV) that typically presents with a variable tetrad of symptoms. HSP if often preceded by respiratory tract infections, vaccinations, drugs or malignancies. During the recent COVID-19 pandemic multiples cases of HSP have been described after both infection and vaccination for SARS-CoV2. This study aims to perform a systematic review of literature and describe an additional complicated case of de-novo HSP appeared after the administration of the third dose of a mRNA-SARS-CoV2 vaccination.

**Methods:**

Electronic bibliographic research was performed to identify all the original reports describing cases of de-novo HSP or IgAV appeared after respiratory infection or vaccine administration for SARS-CoV2. We included all case series or case reports of patients who respected our inclusion and exclusion criteria.

**Results:**

Thirty-eight publications met our pre-defined inclusion criteria, for an overall number of 44 patients. All patients presented with palpable purpura variable associated with arthralgia, abdominal pain or renal involvement. Increased levels of inflammation markers, mild leukocytosis and elevated D-dimer were the most common laboratory findings. Up to 50% of patients presented proteinuria and/or hematuria. Almost all skin biopsies showed leukocytoclastic vasculitis, with IgA deposits at direct immunofluorescence in more than 50% of cases.

**Conclusions:**

Our results suggest that the immune response elicited by SARS-CoV2 vaccine or infection could play a role in the development of HSP. Current research suggests a possible role of IgA in immune hyperactivation, highlighted by early seroconversion to IgA found in some COVID-19 patients who develop IgA vasculitis.

## Case report

**Fig. 1:**
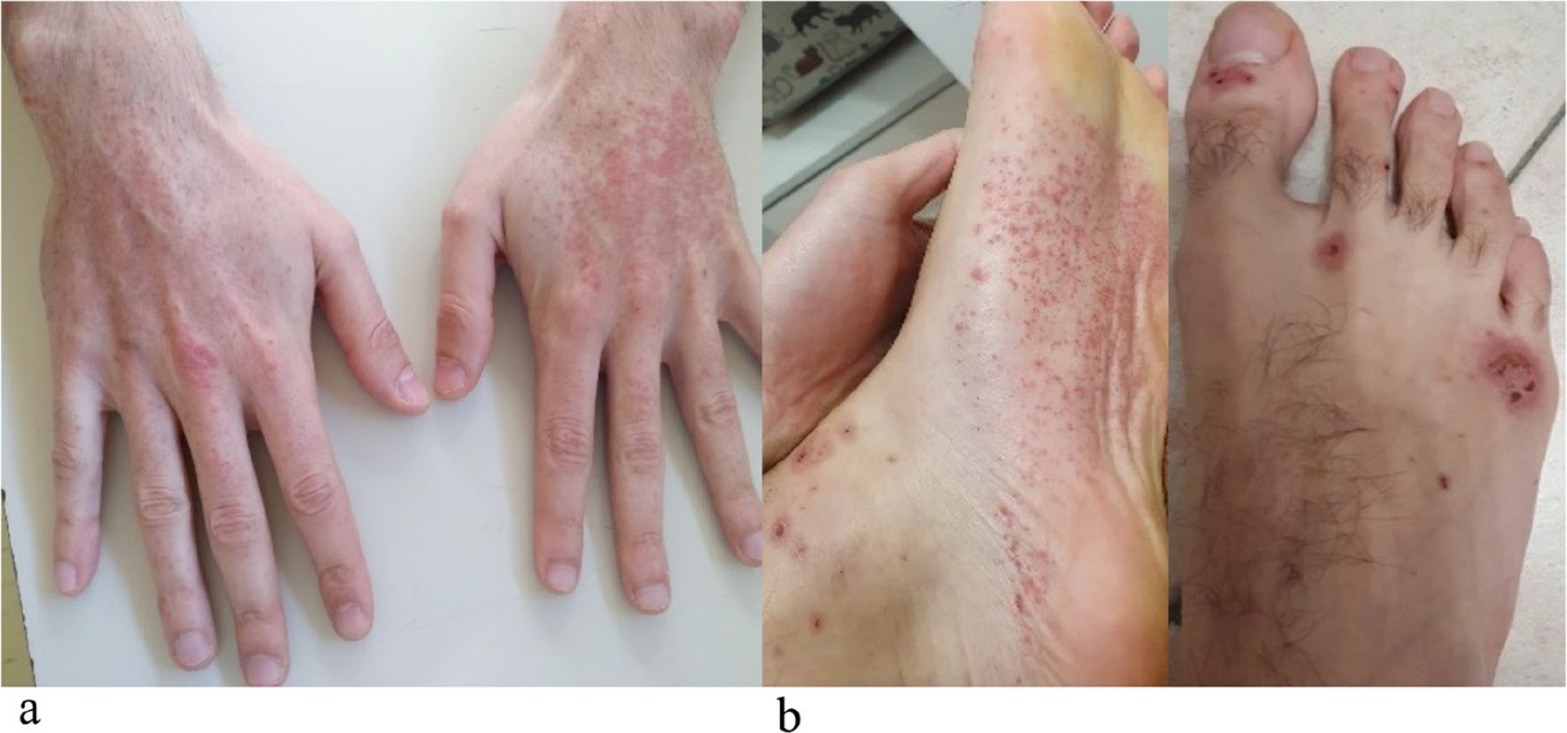
**a** Multiple purpuric lesions. The rash was palpable, non-pruritic, non-blanching; **b** Patient’s feet rash, characteristic of Henoch–Schonlein purpura

A Caucasian 26-year-old male with celiac disease since the age of 6 presented to Emergency Room with healing papules on his feet and multiple, discrete to confluent palpable purpura with few central vesicles distributed symmetrically in lower limbs, auricles, elbows and hands, accompanied by burning pain (Fig. 1). The rash was associated with arthralgia of knee and small joints of hands and feet, without joint swelling, and epigastric burn. He followed a strict gluten-free diet since the diagnosis and serology for celiac disease maintained always negative. His previous medical history was otherwise unremarkable, and he referred not to take any medications at home.

The patient developed these lesions five days after the administration of the third dose of an mRNA SARS-CoV2 vaccination.

Laboratory investigations revealed normal blood cells count, electrolytes, renal and liver functions, and coagulation profile. Markers of inflammation were slightly increased. Tumor-markers were normal, as were viral serologies, autoantibodies, except for anti-nuclear antibodies with low titer, lupus anticoagulant, and anti-Cardiolipin IgG with very low titer, 20.5/20 U/ml. The nasopharyngeal swab for SARS-CoV2 resulted negative.

Absence of proteinuria in 24 h urine collection suggested no kidney involvement.

Skin biopsy of the lesion on the left leg revealed a leukocytoclastic vasculitis, with IgA deposits showed at direct immunofluorescence.

The clinical presentation was consistent with EULAR/ PRINTO/PRES classification criteria for HSP [1], thereby, diagnosis of Henoch–Schonlein purpura was made.

The patient was treated with oral prednisone 1 mg/Kg for two weeks, with rash improvement; at the steroid tapering, new skin lesions appeared, therefore, azathioprine was added. After two more weeks therapy, due to mild increase in amylase and lipase, and absence of clinical response, azathioprine was replaced by cyclosporine. This time skin lesions gradually improved until complete resolution. Cyclosporine was maintained at a stable dosage for about 3 months and subsequently interrupted without symptoms relapse.

## Introduction

Immunoglobulin A (IgA) vasculitis (IgAV) or Henoch–Schonlein purpura (HSP) is an IgA mediated systemic small-vessel vasculitis disorder that arises most commonly in childhood. It affects about 10 to 20 children < 17 years of age per 100.000 per year. Over 90% of patients have less than 10 years, with a peak incidence of 70 per 100.000 per year in children between the ages of 4 and 6 years; males are usually more affected that females (M/F = 1.2/1) [2]. Although adult cases of IgA vasculitis are less frequent, adults commonly have significantly worse renal outcomes than children do. Among adults, the incidence is 3.4 to 14 cases per million per year [3]. IgAV occurs primarily in winter, autumn and spring but rarely in summer months, maybe due to the associations with infections [4, 5].

HSP is a self-limiting multiorgan disorder that typically presents with a variable tetrad of symptom, including palpable purpuric rash, arthralgia, abdominal pain and renal involvement.

The diagnosis of HSP is clinically made and is based on the European League Against Rheumatism (EULAR)/Paediatric Rheumatology International Trials Organisation (PRINTO) [1] /Paediatric Rheumatology European Society (PRES) revised classification criteria developed in 2010. They consist of mandatory and supportive criteria: the mandatory one is the presence of palpable purpura in the absence of thrombocytopenia, while the supportive criteria involve at least one of the following: acute onset of diffuse abdominal pain, acute onset of arthralgia or arthritis, histopathological evidence of leukocytoclastic vasculitis or proliferative glomerulonephritis with IgA deposits and renal involvement documented by proteinuria or hematuria.

HSP is often preceded by upper respiratory tract infections (commonly caused by parainfluenza viruses, human Parvovirus B19 and streptococcal species), vaccinations, food allergies, insect bites, drugs or malignancies, although the exact etiology of this disease is still unknown [6].

During the recent pandemic of Coronavirus disease-19 (COVID-19), caused by severe acute respiratory syndrome coronavirus-2 (SARS-Cov2), a single-strand RNA virus from beta-Coronaviridae family, some cases of HSP have been described after the virus infection [7]. SARS-Cov2 often affects the respiratory system, with presentation ranging from a mild cold-like illness to severe and potentially fatal pneumonia with subsequent acute distress respiratory syndrome or septic shock [8]. COVID-19 has also been linked to several extrapulmonary manifestations, including vasculitis, such as multisystem inflammatory disease of children predominantly in pediatric patients [9], urticarial vasculitis and ANCA-associated vasculitis in adults [10].

Nowadays with the unprecedented volume of vaccinations against Sars-Cov2 worldwide, some cases of HSP following this vaccination are being reported, suggesting that immune activation induced by the vaccine could trigger IgA vasculitis. Anyway, the role of COVID‐19 in precipitating IgA vasculitis is unconfirmed [7].

Here, we report a case of HSP following COVID-19 mRNA vaccination and perform a systematic literature review to collect all reported cases of IgA systemic vasculitis developed after both COVID-19 infection and COVID-19 vaccination.

## Materials and methods

This systematic review was conducted, when possible, according with the Preferred Reporting Items for Systematic Reviews and Meta-Analyses guidelines [11].

### Eligibility criteria

All the original reports which described the insurgence of de novo Henoch–Schonlein Purpura or systemic IgA vasculitis with skin involvement after SARS-CoV2 infection, documented by a nasopharyngeal swab, or Covid-19 vaccination were considered for inclusion. Eligible patients received a diagnosis of HSP according to the EULAR/PRINTO/PRES classification criteria for HSP [1]; patients with cutaneous manifestations after COVID-19 immunization who did not respect these criteria were not included. We excluded studies where the IgA vasculitis occurred after a “supposed” Covid-19 infection, based on typical symptoms or SARS- CoV2 IgG positivity, but not confirmed by a nasopharyngeal swab. We also excluded studies evaluating relapse of pre-existing HSP or IgA vasculitis after Covid-19 exposure or immunization, as well as those including patients who developed IgA glomerulonephritis without systemic involvement. We did not include non-original reports or animal model studies. Due to the scarcity of studies with adequate sample sizes, we included all the reported case series and case reports, without year-span limits. In the search filter we did not use any language restriction.

### Information sources and search strategy

A literature search was performed using the following electronic databases: PubMed, Google Scholar, Web of Science (ISI). The last search was run on 01 May 2022. The terms “Henoch–Schonlein Purpura” OR “HSP” OR “IgA vasculitis” were matched with the following words: “Covid-19” OR “Sars-Cov2 vaccine” OR “Sars-Cov2 vaccination” OR “Sars-Cov2 infection” OR “Covid-19 vaccine” OR “Covid-19 vaccination”. All the terms were searched both as keywords and Medical Subject Headings (MeSH). We hand-searched the bibliographies of relevant (according to titles and abstracts) articles to provide additional references.

### Study selection

Titles and abstracts were independently assessed by two reviewers (F. D. V. and S. E.) to determine the eligibility of the studies. Both investigators checked the fulfillment of inclusion and exclusion criteria; in the case of doubt, the full text of the articles was retrieved and reviewed. A third author (G. C.) arbitrated in all the cases of a lack of agreement.

### Data collection process

Data from eligible studies were independently extracted by two reviewers (F. D. V. and S. E.), then cross-checked. Discrepancies were rectified by consensus. If the article grouped patients from a previous study and newly enrolled ones, only the latter were considered. In the case of mixed cohorts, only data regarding patients who respected the eligibility criteria were included in the analysis.

## Results

### Characteristics of included studies

After literature search and review of titles and abstracts, 38 articles met our eligibility criteria. We divided all the selected articles into two groups and then analyzed separately their characteristics: the first one was composed by all the selected cases of HSP following SARS-CoV2 infection, while the second one included all the cases of HSP occurring after SARS-CoV2 vaccination. HSP diagnosis has always been made according to the EULAR/PRINTO/PRES criteria [1]. All of them were case series or case reports published between 2020 and 2022. A list of all the excluded articles and reasons for the exclusion, as well as the PRISMA diagram of study selection will be available from the corresponding author upon request. In the final analysis we included also our case report. Table 1 summarizes findings from all studies reporting the onset of systemic IgA vasculitis after infection by Covid-19, while Table 2 contains findings from all studies describing the onset of IgA vasculitis after SARS-CoV2 vaccine administration.

**Table 1:** De novo Henoch–Schoenlein Purpura after SARS-CoV2 infection

Author (Reference)	Age, Sex, Race	Comorbilities	Medications taken	Symptoms	Days after SARS-Cov2 Test Positive	Skin involvement (0 = No; 1 = Yes)	Gastrointestinal tract involvement (0 = No; 1 = Yes)	Joint involvement (0 = No; 1 = Yes)	Renal Involvement (0 = No; 1 = Yes)	Laboratory findings (abnormalities)	Microscopic Hematuria/Proteinuria (0 = no Hematuria/Proteinuria; 1 = Hematuria; 2 = Proteinuria; 3 = Hematuria and Proteinuria)	Skin biopsy findings	Direct immunofluorescence (DIF) for IgA (0 = Negative; 1 = Positive)	Renal biopsy findings	Direct immunofluorescence (DIF) for IgA (0 = Negative; 1 = Positive)	Treatment
Sandhu S. et al. 2020 [12]	22, M	NR	None	Fever Abdominal pain Vomiting Painful swelling of ankle and wrist joints Multiple red lesions simmetric over all extremities	2	1	0	1	1	Mild increase of AST and ALT	2	Leukocytoclastic vasculitis	0	Focal endocapillary proliferative IgA nephropathy with mesangial granular deposits of IgA	1	Dexamethasone
AlGhoozi D.A. et al. 2021 [13]	4, M	None	NR	Orthostatic pruritic maculopapular rash Mild oedema in the ankles and ankle pain	37	1	0	1	0	Unremarkable	0	NA	NA	NA	NA	Paracetamol
Borocco C. et al. 2021 [14]	13, F	Panhypopituitarism	Thyroid hormones Growth hormones Desmopressin Hydrocortisone	Infiltrated purpuric and ecchymosis lesions on lower limbs and buttocks Ankle edema Low-grade fever Intense abdominal pain	Same time	1	1	0	0	Mild leukocytosis Increased CRP Mild increased IgG Increased IgA Positive PCR for EBV	0	NA	NA	NA	NA	None
Asiri A. et al. 2022 [7]	4, M	Eczema	None	Obstructive jaundice Small pinpoint rash on the feet Insect bites in flexor and extensor surfaces	Same time	1	0	1	0	Unremarkable	NA	NA	NA	NA	NA	Prednisolone
Asiri A. et al. 2022 [7]	2, M	None	NR	Left-hand swelling, pain and decrease in the range of motion Orthostatic purple rash Painful defecation associated with streaks of blood, bloody stool Severe lower abdominal pain (intussusception) Fever Bilateral wrist pain, swelling Right knee joint swelling and functional impotence	Same time	1	1	1	0	Unremarkable	NA	NA	NA	NA	NA	Prednisolone
Asiri A. et al. 2022 [7]	4, M	None	None	Wrists, knees, and left ankle joints swelling, pain, functional impotence Epigastric intermittent pain Diarrhea, blackish stool Orthostatic nonpruritic rash	Same time	1	1	1	0	Increased D-Dimer Positive occult blood in stool Increased total protein and albumin levels	NA	NA	NA	NA	NA	Prednisolone
Allez M. et al. 2020 [15]	24, M	Crohn's disease	Adalimumab	Orthostatic palpable purpura Swelling on the left hand Severe arthralgia Abdominal pain	9	1	1	1	0	Increased CRP Increased D-dimer Increased Fibrinogen Increased serum IgA levels	NA	Leukocytoclastic vasculitis	1	NA	NA	Low molecular weight heparin Intravenous steroids
Jacobi M. et al. 2021 [16]	3, M	Surgically corrected Hirschsprung disease	Metronidazole	Orthostatic purpuric rash Abdominal pain Nonbilious emesis	2	1	1	0	0	Microcytic anemia Mild thrombocytosis Mild metabolic acidosis at venous blood gases	NA	NA	NA	NA	NA	Antibiotic therapy Methyl prednisone
Barbetta L. et al. 2021 [17]	62, M	NR	Bisoprolol Telmisartan Statin Basal-bolus insulin SGLT2-inhibitor	Dyspnea Fever Orthostatic purpuric lesions with raised papules Acute abdominal pain Vomiting Haematochezia	10	1	1	0	1	Unremarkable	3	Leukocytoclastic vasculitis	1	NA	NA	Continuous positive airway pressure (CPAP) Hydroxychloroquine Lopinavir/ritonavir Levofloxacine Methylprednisolone
Ziyara R. et al. 2022 [18]	12, M	None	None	Abdominal pain Loose stools Nausea Purpuric rashes in bilateral lower extremities Painful swellings of the small joints in the hands bilaterally	1, relapse 2 months after	1	1	0	NR	Mild leukocytosis Increased CRP	NA	NA	NA	NA	NA	Oral prednisolone
Li, N.L. et al. 2021 [19]	30, M	None	None	Fever Runny nose, cough Diarrhea Abdominal pain Painful purpuric rash to his lower extremities, distal upper extremities, and trunk prompting Arthralgia	Same time	1	1	1	1	Increased CRP Positive D-dimer Mild prolongation of INR and aPTT Increased ALP and GGT	3	Leukocytoclastic vasculitis	0	Crescentic and segmentally necrotizing IgAN with focal endocapillary hypercellularity Focal tubular atrophy and interstitial fibrosis	1	Prednisolone Losartan
Kumar G. et al. 2021 [20]	13, M	None	None	Orthostatic palpable and non-pruritic rash	28	1	0	0	1	Increased ESR Mildly elevated IgA	1	Leukocytoclastic vasculitis	0	NA	NA	Oral prednisolone
Jedlowski P.M. et al. 2021 [21]	70, M	NR	NR	Diarrhea Bilateral symmetrical arthralgias of the wrists, ankles, and knees Abdominal pain Purpuric rash on the bilateral lower extremities, buttocks, and abdomen	7	1	1	1	1	Increased ESR and CRP Acute kidney injury	3	Leukocytoclastic vasculitis	1	Mesangial hypercellularity Focal/mild endocapillary hypercellularity Tubular atrophy, interstitial fibrosis and lymphocytic tubulitis but without crescent	1	Dexamethasone (8 days) Methylprednisolone (3 days) Prednisone (1 month)
Serafinelli J. et al. 2021 [22]	10, F	None	None	Purpuric lesions Erythema pernio-like (feet and lower limbs) Nephritic-nephrotic syndrome (after 4 weeks)	Same time	1	0	0	1	Mild increase of serum creatinine Hypoalbuminemia	3	NA	NA	Diffuse and segmental mesangial-proliferative glomerulonephritis Fibrocellular crescents, interstitial fibrosis, and diffuse segmental glomerular sclerosis	1	Intravenous methylprednisolone and oral prednisone Cyclophosphamide Mycophenolate mofetil ACE-inhibitors
Hoskins B et al. 2021 [23]	2, M	None	None	Abdominal pain Hematochezia Purpuric lesions	Same time	1	1	0	0	Elevated D-dimer Increased CRP and ESR	NA	NA	NA	NA	NA	Intravenous steroid
Riscassi S. et al. 2021 [24]	3, M	None	None	Fever Orthostatic urticaria wheals	NR	1	0	0	0	Leukocytosis Increased CRP	NA	NA	NA	NA	NA	Antibiotic therapy Histaminic therapy
Suso A. S. et al. 2020 [25]	78, M	Essential hypertension Dyslipidemia Moderate aortic valve stenosis Bladder cancer	Losartan	Wrist arthritis and lower limb purpura	34	1	0	1	1	Increased serum creatinine Hypoalbuminemia	3	Cutaneous vasculitis	1	Segmental mesangial expansion with hypercellularity Epithelial crescents	NA	Methylprednisolone pulses Rituximab
Imen Y. et al. 2022 [26]	57, F	Hypertension	NA	Non‐necrotic purpura (1–2 cm) on the legs and lower back	13	1	0	0	0	Unremarkable	NA	Leukocytoclastic vasculitis	NA	NA	NA	None
Mustafa O. Al Haji et al. 2021 [27]	13, F	None	None	Lower limb skin rash Fever Abdominal pain Chills Hematuria	14	1	1	0	0	Increased CRP and ESR	0	Orthokeratosis in the epidermis Leukocytoclastic vasculitis	NA	NA	NA	Ceftriaxone Methylprednisolone Omeprazole
Soleiman-Meigooni S. et al. 2021 [28]	21, M	None	None	Fever Myalgia Dyspnea Dry cough Orthostatic purpuric rash	8	1	0	0	1	Positive HLA-B27	2	Leukocytoclastic vasculitis	NA	NA	NA	Ceftriaxone Pantoprazole Azithromycin Lopinavir Ritonavir Dexamethasone
Camprodon Gómez M. et al. 2020 [29]	29, M	None	Pre-exposure prophylaxis for 2 months	Orthostatic purple palpable papules Muscular pain Colic abdominal pain with diarrhoea and a single episode of hematochezia	28	1	1	0	0	Elevated D-dimer Increased ESR	0	Leukocytoclastic vasculitis	0	NA	NA	Corticosteroids
Nakandakari Gomez M.D. et al. 2021 [30]	4, M	None	None	Dry cough and rhinorrhea Epigastric abdominal pain Orthostatic purplish red punctate and other maculopapular lesions	8	1	1	0	1	Thrombocytosis Normochromic normocytic anemia Decreased values of total proteins Hypoalbuminemia Prolonged aPTT Increased ESR Strongyloides stercoralis	1	NA	NA	NA	NA	Ceftriaxone Metronidazole Ivermectin Omeprazole Dexamethasone, than prednisone
Bekhit Osama E. et al. 2021 [31]	5, F	Atopic dermatitis	Local steroids Emollients	Fever Generalized muscular pain Confluent palpable purple red papular rash on both ankles Joints pain	Same time	1	0	1	0	Leukocytosis Anemia Increased CRP Increased IgA	0	NA	NA	NA	NA	Antibiotics (ampicillin, sulbactam, and ceftriaxone) Intravenous fluids Antipyretic analgesics Prednisolone

*F* female, *M*, male, *CRP* C-reactive protein, *ESR* erythrocyte sedimentation rate, *ALP* alkaline phosphatase, *GGT* gamma glutamyl transferase, *ALT* alanine amino transferase, *AST* aspartate amino transferase, *INR* international normalized ratio, *aPTT* activated partial thromboplastin time, *ANA* anti‐nuclear antibodies, *RF* rheumatoid factor, *IgG* immunoglobulin G, *IgA* immunoglobulin A, *NR* not reported, *NA* not available.

**Table 2:** De novo Henoch–Schoenlein Purpura after SARS-CoV2 vaccine administration

Author (Reference)	Age (years), Sex	Comorbilities	Medications taken	Type of vaccine administrated	Days after SARS-Cov2 infection/vaccination	Symptoms	Skin involvement (0 = No; 1 = Yes)	Gastrointestinal tract involvement (0 = No; 1 = Yes)	Joint involvement (0 = No; 1 = Yes)	Renal Involvement (0 = No; 1 = Yes)	Laboratory findings (abnormalities)	Microscopic hematuria/Proteinuria (0 = no Hematuria/Proteinuria; 1 = Hematuria; 2 = Proteinuria; 3 = Hematuria and Proteinuria)	Skin biopsy findings	Direct immunofluorescence (DIF) for IgA (0 = Negative; 1 = Positive)	Renal biopsy findings	Direct immunofluorescence (DIF) for IgA (0 = Negative; 1 = Positive)	Treatment
Our case	26, M	Caeliac disease	Strict gluten-free diet since the year of diagnosis	Moderna (III dose)	5	Discrete to confluent palpable purpura with few central vesicles distributed symmetrically in the lower limbs, auricles, elbows and hands accompanied by burning pain Arthralgia of knee and small joints of hands and feet Epigastric burn	1	1	1	0	Increased CRP and ESR Positive ANA (low titer) Positive lupus anticoagulant (LAC) and anti-Cardiolipin IgG (very low titer)	0	Leukocytoclastic vasculitis	1	NA	NA	Oral prednisone Azathioprine Cyclosporine
Naitlho A. et al. 2021 [32]	62, M	Osteosarcoma Intercostal shingles Tonsillectomy Mild COVID-19 infection in October 2020	None	ChAdOx1 nCoV-19 AZD1222 (Vaxzevria—AstraZeneca)	8	Orthostatic petechial purpuric rash Polyarthralgia (knees and ankles)	1	0	1	0	Increased CRP Increased D-dimers Positive ANA and RF	1	NA	NA	NA	NA	Prednisone
Sirufo M.M. et al. 2021 [33]	76, F	None	Calcifediol	ChAdOx1 nCoV-19 AZD1222 (Vaxzevria—AstraZeneca)	7	Orthostatic purpuric rash Coxalgia Macrohaematuria	1	0	1	1	Increased CRP and ESR	1	NA	NA	NA	NA	Paracetamol Deflazacort
Hines A.M. et al. 2021 [34]	40, F	Headaches Hashimoto’s thyroiditis		Pfizer‐BioNTech BNT16B2b2 (II dose)	20	Orthostatic purpuric rash	1	0	0	0	Mild leukocytosis	NA	NA	NA	NA	NA	None
Sugita K. et al. 2022 [35]	67, F	Hypertension	NR	Pfizer-BioNTech (II dose)	5 h	Erythematous maculopapular rash on left leg	1	0	0	1	Increase of serum creatinine (eGFR) 52.6 ml/min/1.73m2	3	Leukocytoclastic vasculitis	1	Mesangial or endocapillary proliferative Necrotizing cellular crescent formation	1	Cyclophosphamide
Nakatani S. et al. 2022 [36]	47, M	Hypertension Hyperuricemia	Azilsartan Amlodipine Febuxostat	mRNA-1273 COVID-19 (II dose)	15	Palpable purpuric papules on legs and feet	1	0	0	1	Unremarkable	3	Perivascular dermatitis with mixed inflammation, including lymphocytes, neutrophils, and related nuclear dust	NA	Severe crescentic glomerulonephritis	1	Methylprednisolone
Grossman M.E. et al. 2021 [37]	94, M	Chronic atrial fibrillation Bioprosthetic aortic valve replacement Prostatectomy, Hypothyroidism Chronic anemia	Apixaban Cyanocobalamin Dutasteride Ferrous sulfate Folic acid Furosemide Levothyroxine Omeprazole Pravastatin Angiotensin‐converting enzyme inhibitor Sertraline	mRNA‐1273 (II dose)	10	Orthostatic palpable purpura	1	0	0	1	Increase of serum creatinine Increased erythrocyte sedimentation rate Positive ANA Anemia	3	Leukocytoclastic vasculitis	1	NA	NA	Prednisone
Mohamed M. et al. 2021 [38]	50, M	Seasonal allergy Mild COVID-19 infection 4 months before	none	BNT162b2 (mRNA), Pfizer (II dose)	1	Orthostatic violaceous nonblanching papules and blisters Myalgias	1	0	1	1	Increased CRP and ESR Acanthocytes	2	Leukocytoclastic vasculitis	1	Mesangial hypercellularity with IgA granular diffuse deposits Mild glomerular sclerosis	1	Prednisone
Badier L. et al. 2021 [39]	72, M	Hypertension Myocardial infarction Type 2 diabetes mellitus Obesity Asthma	Irbesartan Hydrochlorothiazide Amiloride Bisoprolol Lercanidipine Rilmenidine Simvastatin Acetylsalicylate acid Metformin Rabeprazole Inhaled combination of formoterol and budesonide	ChadOx1 nCoV-19-Oxford-AstraZeneca (I dose)	15	Orthostatic vascular purpura Arthralgia of the ankles, knees and shoulders	1	0	1	0	Increased CRP	0	Leukocytoclastic vasculitis	1	NA	NA	Prednisone
Iwata H. et al. 2021 [40]	70, F	Rheumatoid arthritis Chronic renal failure	Adalimumab Hemodialysis	Pfizer-BioNTech (II dose)	2	Orthostatic palpable purpura	1	0	1	0	Increased CRP Increased IgG and IgA	NA	Leukocytoclastic vasculitis	1	NA	NA	None
Nishimura N. et al. 2022 [41]	30, M	NR	none	Pfizer-BioNTech BNT16B2b2 mRNA vaccine (II dose)	5	Orthostatic palpable purpura Fever Abdominal pain Swelling of the left knee	1	1	1	0	Mild leukocytosis Increased CRP Positive antinuclear antibody	3	Leukocytoclastic vasculitis	0	NA	NA	Prednisone
Nishimura N. et al. 2022 [41]	22, M	Recurring tonsillitis	none	Phizer-BioNTech BNT16B2b2 (I dose)	6	Rash on the lower limbs Arthralgia	1	0	1	0	Mild leukocytosis Increased CRP Normal Anti-streptolysin O antibody (ASO) Normal anti-streptokinase antibody (ASK)	NA	Leukocytoclastic vasculitis	1	NA	NA	Prednisolone
Abdelmaksoud A. et al. 2022 [42]	17, F	NR	NR	Phizer-BioNTech BNT16B2b2 (I dose)	10	Palpable purpura on both arms and legs	1	0	0	0	Unremarkable	NA	Leukocytoclastic vasculitis	1	NA	NA	Systemic antihistamine Local steroid
Abdelmaksoud A. et al. 2022 [42]	48, M	NR	NR	Phizer-BioNTech BNT16B2b3 (II dose)	4	Palpable purpura on both legs	1	0	0	0	Unremarkable	NA	Leukocytoclastic vasculitis	NA	NA	NA	Systemic antihistamine Local steroid
Roy R.M. et al. 2022 [43]	60, F	Hypertension Hypothyroidism	Telmisartan L-thyroxine	Covishield vaccine (a nonreplicating adenovirus vector vaccine) (I dose)	8	Orthostatic palpable purpuric rash	1	0	0	1	Increased CRP and ESR	1	Immune complex vasculitis with immunoglobulin M, C3, and fibrinogen deposits in the vessel wall	0	NA	NA	Colchicine Pantoprazole
Bostan E. et al. 2021 [44]	33, M	None	None	Messenger ribonucleic acid (mRNA) COVID‐19 vaccine	3	Erythematous macules and palpable papules on the legs, forearms, and belly	1	0	0	0	Unremarkable	NA	Hyperkeratosis in the epidermis Leukocytoclastic vasculitis	1	NA	NA	Topical mometasone furoate
Liang I. et al. 2021 [45]	62, F	NA	NA	ChAdOx1 nCoV-19 vaccine (AstraZeneca)	7	Bilateral lower limb petechial rash Headache Myalgia Large joint arthralgias	1	0	1	0	Increased CRP	0	Leukocytoclastic vasculitis	0	NA	NA	Prednisolone
Fiorillo G. et al. 2022 [46]	71, F	Fibrocystic mastopathy Arterial hypertension	Atenolol	Vaxzevria COVID-19 vaccine (AstraZeneca) (II dose)	5	Purpuric macules and papules on both legs	1	0	0	0	Mild leukocytosis Increased D-dimer level Increased CRP Mild impairment of C3 and C4 Mild increase of RF	NA	Leukocytoclastic vasculitis Linear and granular deposition of IgM within small vessels	0	NA	NA	Prednisone
Oniszczuk J. et al. 2021 [47]	55, F	Diabetes Sleep apneoa Asthma	NR	ChAdOx1 nCoV-19 (AstraZeneca) (II dose)	2	Flu-like syndrome Orthostatic palpable purpura Arthralgia of the knees and ankles Diarrhea	1	1	1	1	Increased CRP	2	Leukocytoclastic vasculitis	1	Proliferative IgA nephropathy, with segmental endocapillary hypercellularity and cellular crescent	1	Refused therapy
Hashizume H. et al. 2022 [48]	16, F	None	NR	Pfizer-BioNTech BNT16B2b2 mRNA (I dose)	2	Pin-head-sized papules on both legs Recurrent epigastric pain Slight left knee joint swelling	1	1	1	1	Increased total IgG	3	Leukocytoclastic vasculitis	1	NA	NA	Diphenyl sulfone

*F* female, M male, *CRP* C-reactive protein, *ESR* erythrocyte sedimentation rate, *ANA* anti‐nuclear antibodies, *RF* rheumatoid factor, *IgG*, Immunoglobulin G, *IgA* immunoglobulin A, *NR* Not reported, *NA* not available.

Tables 1 and 2 show data extracted from each primary study.

### Characteristics of patients

A total of 43 patients developed IgAV after SARS-CoV2 exposure, of whom 23 after the natural virus infection (I Group) and 20 after Covid-19 vaccination (II Group), including our case.

Among the first group 18 patients were male, with a M/F ratio of 3.6/1, as opposed to the slight male sex predilection (from 1.2 to 1.8/1) seen in others IgAV cases reported previously [5]. Patients aged from 2 to 78 years, with a middle age of 21.09; pediatric patients (age less than 18) represented 14 of 23 (61%) cases, with patients aged from 4 to 6 years comprising five of these. The remainder occurred in adult patients (9/23, 39%). Compared to previously reported cases of HSP, characterized by a peak of incidence (over 90% of total cases) in pediatric populations, SARS-CoV2 associated IgAV occurred frequently in adult patients (39%). Time between the first COVID‐19 symptom onset or the first positive nasopharyngeal swab and the development of cutaneous purpura ranged from 2 to 37 days, with a middle time of 14.3 days.

In the group of patients developing IgAV after COVID-19 vaccination, which included also our patient, males were 10 among 20 patients, with a M/F ratio of 1/1; in contrast with the usual prevalence of HSP in males.

The age of cases ranged from 16 to 94 years, with a middle age of 50.9 y.o., with pediatric patients which represented only two of 20 (10%) cases. This result anyway could partly be explained by the higher vaccination rate for COVID-19 in the adult population, compared with pediatric population. The majority of cases (13 of 20, 65%) received an mRNA SARS-CoV2 vaccine, while the remaining 7 patients were administrated with a nonreplicating adenovirus vector vaccine. Five of 20 cases developed IgAV after the I dose of the vaccination, 10 cases after the second administration and one case after the third. Time from dose administration and the development of IgAV ranged from five hours to 20 days.

### Clinical presentation

All selected cases of COVID-19-associated IgAV presented with palpable purpura without thrombocytopenia, and at least one more criteria among those required for HSP diagnosis, according to the EULAR/PRINTO/PRES criteria [1].

In the first group 13 of 23 cases (57%) presented also gastrointestinal symptoms, 10 of 23 (43%) had a joint involvement and 9 of 23 cases (39%) presented a kidney injury. Globally, these results reflect the percentages of organs involvements observed in other IgAV reported cases, except from the lower rate of arthralgias detected in COVID-19-associated-IgA (43%) respect to typical HSP (up to 84%) [10]. Gastroenterological symptoms tended to be more frequent in pediatric patients compared to adult; indeed, in our cases only one patient, a 2-year-old child, was finally diagnosed with intestinal intussusception, which is the most frequent gastrointestinal complication in HSP patients [49].

On the contrary, acute kidney injury and proteinuria occurred almost exclusively in adult patients, indeed only three cases of hematuria and nephrotic range proteinuria were described among children. There were no differences in arthralgias frequency between adults and infants.

In the group of patients who developed HSP after COVID-19 vaccination only 4 patients (20%) had gastrointestinal symptoms, while 11 of 20 cases (55%) complained arthralgias and 8 of 20 (40%) had a kidney involvement. Among the 2 infants present in this group, one showed only a cutaneous involvement, while the second presented also articular, gastrointestinal and renal symptoms.

Globally, all the reported cases did not show the involvement of other organs occasionally involved in IgAV, such as central and peripheral nervous system, eyes and urologic involvement.

### Laboratory and histopathological findings

In the first cohort increased levels of C-reactive protein and/or erythrosedimentation rate was the most diffused laboratory findings at presentation (12 of 23 cases, 52%), followed by increased D-dimer (5/23, 22%), leukocytosis (4/23, 17%) and hypoalbuminemia (3/23, 13%). Only two patients had increased hepatic enzymes or cholestasis, while four showed increase of total serum IgA. One patient tested positive for occult blood in stool and one developed acute kidney injury with creatinine from baseline 0.8 mg/dl to 3.8 mg/dl, while other two showed only a mild increase of serum creatinine. All patients had normal platelet count and normal coagulation profile, except one who showed mild increase of INR and aPTT.

When urine tests were performed, 7 cases presented proteinuria, 5 of whom had also hematuria. Only two patients developed hematuria without proteinuria. Among infant patients, three of them showed a renal involvement with hematuria, one of whom presenting also proteinuria.

11/23 (48%) patients were tested with skin biopsy; all except one showed histopathological findings compatible with leukocytoclastic vasculitis. Direct immunofluorescence for IgA was performed in 8/11 cases, resulting positive in 4/8 (50%). In 5 patients also kidney biopsy was performed, due to the laboratory finding of proteinuria/hematuria. At the histopathological analysis 4/5 biopsies revealed the presence of mesangioproliferative nephritis with IgA deposits (IgAN), detected with direct immunofluorescence for IgA, variable associated with epithelial crescents and focal endocapillary hypercellularity. In one case DIF was not performed, and the histopathological analysis of kidney biopsy showed segmental mesangial expansion with hypercellularity and epithelial crescents.

Among patients in II Cohort 12 of 20 cases (60%) were characterized by an increase of serum inflammatory markers, particularly CRP. All patients had normal coagulation profile and blood cells count, except from mild leukocytosis in 4/20 (20%) patients. Other common laboratory findings were the presence of both proteinuria and hematuria in 5 over 13 patients tested (25% of total cases), isolated proteinuria in 2/13 (10% of total cases) and isolated hematuria in 3/13 (15% of total cases). In three patients, Anti-nucleus antibodies resulted positive, while two patients presented an increase of Rheumatoid Factor. Serum total IgA had been tested only in four patients, and only one showed increased total IgA levels. Skin biopsy was performed in 17/20 patients: at the histopathological examination, almost all cases resulted compatible with leukocytoclastic vasculitis. Direct immunofluorescence for IgA has been executed in 15 over 17 skin biopsies, resulting positive in 11/15 (73%) of cases. Two patients showed Immunoglobulin M, C3, and fibrinogen deposits in the vessel wall at the direct immunofluorescence test and histopathological analysis. In 4 patients who presented proteinuria, kidney biopsy was performed. All the exams revealed a mesangioproliferative nephritis with IgA deposits (IgAN) at the direct immunofluorescence test.

### Treatment and prognosis

In the first group, 19/23 (83%) patients were treated with oral or intravenous corticosteroids, especially prednisone and methylprednisolone, leading to clinical improvement and then complete resolution of skin lesions. One patient, due to recurrence of purpura despite steroid therapy, was treated also with Rituximab reaching complete healing of purpuric rash. In another case treatment also with cyclophosphamide, mycophenolate mofetil and ACE-inhibitors was needed due to the significant kidney involvement, not responsive to methylprednisolone. The remaining 4 patients did not require any immunosuppressive therapy for the complete resolution of purpura.

In second group, 11/20 (55%) patients required a corticosteroid therapy, particularly with prednisone or methylprednisolone for the complete healing of skin lesions. Seven patients undergone a restitutio ad integrum of the rash only with topical treatment such as local steroid or topical mometasone furoate or even without any treatment. Only two patients in the second group required more aggressive therapy for the resolution of IgA vasculitis: the first one (our case) was treated with azathioprine and, then, cyclosporine, while the second received a therapy with cyclophosphamide. Finally, one patient improved after diphenyl sulphone treatment and another with colchicine.

## Discussion

In the last years, the ability of SARS-CoV2 to affect almost every human organ became always clearer, thus causing renal, cardiac, cutaneous, psychological, neurological and vascular diseases [50–53]. Various types of vasculitides have been reported with COVID-19 infection [54]. In the case we presented above, the clinical presentation, the personal history of autoimmune disease, like celiac disease, and the pathologic findings suggested that Sars-Cov2 vaccine may have been the trigger for the unmasking of an autoimmunological trait that led to the development of Henoch–Schonlein purpura.

In this review, we discussed 23 cases of de novo HSP occurring after infection by COVID-19 and 20 after SARS-CoV2 vaccination. In the first group reported cases were mostly male, with a middle age of 21.9 years; these data oppose with the usual mild prevalence of HSP in males and with the usual prediction of IgA vasculitis for children between 5 and 7 years old. Globally, these patients presented less articular involvement compared with typical HSP patients who show arthritis/arthralgia in up 84% of cases. On the contrary they showed similar gastrointestinal (57%) and renal involvement (39%); indeed, gastrointestinal symptoms usually are present in approximately one-half of HSP patients, ranging from mild (nausea, abdominal pain, vomiting) to severe findings, such as gastrointestinal hemorrhage, bowel ischemia and necrosis, intussusception and bowel perforation [49]; while kidney involvement had been reported in 20 to 54% of children with IgAV. In the second group, we observed the same incidence rate of HSP between men and women; and the middle age of patients was significantly higher than that of I group. It could probably be explained by the low vaccination rate for SARS-CoV2 observed among infants. Percentage of articular and kidney involvement were almost the same, while the one of gastrointestinal involvement was lower than that of the first group. Overall, most common laboratory findings were increased CRP, mild leukocytosis, especially neutrophilia and elevated D-dimer. These nonspecific findings may reflect inflammation triggered by COVID-19 rather than the vasculitis itself. Despite common cases of IgAV, where serum IgA levels are elevated in 50 to 70 percent of patients [55], in our cases only few patients showed this laboratory findings, but maybe only because in many cases IgA had not been dosed at all.

However, little is known about the possible pathogenetic mechanism responsible of the association between COVID-19 and IgA-mediated systemic vasculitis (Henoch–Schonlein Purpura).

COVID-19 infection is notoriously associated with vascular endothelial injury and organ vasculitis. This could derive both from endothelial cells’ invasion by SARS-CoV2 and from inflammatory reaction derived by the infection. Some mechanisms for viral cells invasion have been hypothesized, such as the role of angiotensin-converting enzyme 2 (ACE2) receptors, scavengers receptor B type 1 (SR-B1) and other cellular wall receptors that allow the entry of the virus into the endothelial cells, thus determining endothelial dysfunction and endothelialitis. Those vascular alterations generate a prothrombotic and proinflammatory milieu leading to excessive thrombin production, inhibition of fibrinolysis and activation of complement pathways. The thrombo-inflammatory state can finally provoke the deposition of microthrombi in small vessels and a microvascular dysfunction [56]. Interestingly, the case reported by Gómez et al. which described the insurgence of HSP one month after symptomatic COVID-19 infection in a 29-year-old man, was characterized by the presence of a positive SARS-CoV2 PCR from a skin biopsy of the patient [29].

Moreover, infection by SARS-CoV2 determines a dysregulation of the immune system response and the consequent cytokine-release syndrome, due to the overactivation of innate immunity in the setting of T-lymphocytes depletion. During the infection an important increase of cytokines like IL-6, interferon γ (IFN-γ) and Tumor Necrosis Factor alpha (TNF α) had been documented. TNF-α, in turn, induces the production of reactive oxygen species which determines further damage to the endothelial cells, causing endothelial dysfunction and inflammation. Indeed, cytokines levels, especially IL-6 levels differ according to the severity of the disease, with higher values in patients with worse prognosis; therefore, they could even be used as biomarker for the prediction of morbidity duration and mortality in infected patients [56].

The critical role of mucosal and systemic IgA in the immunological response to SARS-CoV2 is getting attention only recently. In the case of respiratory infection, the seroconversion day of IgA is 2 days after onset of initial symptoms, while the first seroconversion of IgM and IgG is 5 days after onset. Secretory IgA (sIgA) induce strong mucosal immunity; indeed, it is in part the result of IgA-mediated interactions with pathogenic microorganisms that prevent pathogens adhesion to the cell surface. Moreover, it seems that sIgA are able to induce interleukin (IL)-6, IL-8, monocyte chemoattractant protein-1 and granulocyte–macrophage colony stimulating factor production through normal human lung fibroblasts [57]; besides, they have a synergistic effect with IgG in promoting antibody-dependent cellular cytotoxicity (ADCC) [58]. On the contrary, the role of serum IgA is relatively unexplored. Several studies revealed a both proinflammatory and anti-inflammatory effect of serum IgA in innate immune response and suggested a plausible role of IgA as a trigger for autoimmune disease and immune hyperactivation [59]. Indeed, with COVID-19 cases increasing worldwide, cases of IgA vasculitis are also rising. The most widely accepted possible pathogenesis of IgA vasculitis involves Galactose deficient IgA1 (Gd-IgA1). The mucosal SARS-CoV2 infection may enhance IL-6, IL-1 and TNF production, leading to aberrant glycosylation of IgA1; the increase of Gd-IgA1, together with IgG autoantibodies generation, may result in the immune complex formation and precipitation, with consequent activation of complement cascade and inflammatory processes. In the case reports we selected, the one by Allez M. et al. [15] described the insurgence of HSP in a young patient associated with high levels of serum IgA and with only weak and transitory IgA shown on COVID-19 serologic testing. Similar results have been found by M. El Hachem et al. [60] in children who developed chilblain-like lesions during COVID-19 infection: some of them tested positive only for IgA specific for the S1 domain of the spike protein. This case supports the possible role of IgA as a trigger for inflammation at mucosal and non-mucosal sites. Besides, the strong mucosal immunity IgA mediated developed by some people after COVID-19 infection might impair the triggering of an IgG response, causing a dysregulated hyper-immune activation.

Another supposed pathogenetic mechanism of acute IgA-vasculitis associated to COVID-19 regards a form of type 3 hypersensitivity. Recent studies observed that patients requiring intensive care inappropriately mount a Th2 response against SARS-CoV2, with as main effectors: eosinophils, basophils, mastocytes and B cells. The activation of B cells results in the production of antibodies (humoral immunity), and, presumably, given the high antigen load, a type 3 hypersensitivity reaction takes place with consequent accumulation of antigen–antibody complexes in small vessels. The immune complex deposition determines the subsequent activation of the complement cascade, with the release of complement anaphylatoxins (C3a and C5a). In turn, they stimulate the release of histamine from mast cells and the recruitment of phagocytes, resulting in an acute necrotizing vasculitis with neutrophilic infiltrate, fibrinoid necrosis and karyorrhexis, called “leukocytoclastic vasculitis” [61].

In the mRNA 1237 COVID-19 vaccine trial with 30,420 volunteers, only 2 in the placebo group and 11 in the vaccine group had a macro-purpuric eruption, while no one showed glomerulonephritis [62].

After commercialization, with the unprecedented volume of vaccinations against Sars-Cov2 worldwide, several cases of both IgA vasculitis and nephritis had been reported. These clinical observations raise the possibility of a relationship between vaccinations and stimulation of the immune system leading to autoimmune diseases in predisposed subjects. Vaccines aim to induce a host humoral and cellular immune response to exogenous antigens and to elicit a memory T-lymphocytes response through the years. Usually, vaccines use adjuvants to enhance vaccine immunogenicity. The mRNA vaccines, such as Pfizer-BioNTech BNT16B2b2 or Moderna mRNA-1273, do not require an adjuvant since the mRNA itself can stimulate the innate immune response, promoting immune induction, through pattern-recognition receptors such as Toll-like receptor (TLR) 3, TLR7 or retinoid-inducible gene I [63]. These receptors are expressed by immune cells, such as dendritic cells and macrophages. Various TLRs, including TLR3 and TLR7 had been found upregulated in IgA vasculitis; this suggests their possible involvement in the pathogenesis of the vasculitis. Other mechanisms may also be suggested, such as the molecular mimicry of the SARS-CoV2 spike-protein with some autoantigens. mRNA vaccines are composed of lipid nanoparticles containing the mRNA encoding for the viral spike protein. A case report presented by Obeid et al. [64] showed the production of autoreactive IgA anti-HEp-2 cells after mRNA-1273 vaccine administration in a patient with a history of IgA vasculitis, in remission for over 2 years. Serum tests of the patient made before vaccination did not show any autoreactivity, as well as serum taken from two healthy controls after mRNA 1273 vaccine administration. These observations suggest the capability of mRNA SARS-CoV2 vaccine to induce the production of autoreactive antibodies only in susceptible patients.

A study by Jincan Zan et al. of 2022 [65] investigated the safety of COVID-19 vaccine in patients with IgA nephropathy or IgA vasculitis. It finally involved 367 vaccinated patients and 2 patients developed flare-up events, 3 exhibited > 30% estimated glomerular filtration rate decrease and 3 patients progressed to nephrotic proteinuria within 3 months after vaccination. Among 202 patients investigated with urine tests 3 months before and after vaccine administration, there were no significant differences regarding proteinuria and hematuria, while estimated glomerular filtration showed a mild but statistically significant reduction.

Given these findings and results from recent pharmacovigilance studies, we confirm that COVID-19 vaccine-associated HSP is rare [66, 67], and we cannot rule out a fortuitous association in our patient. However, a possible relationship between these two entities, neither confirmed, nor ruled out should be kept in mind. SARS-Cov2 vaccine administration is, thus, globally safe in IgAN and IgA vasculitis patients, with a low absolute incidence of adverse events. However, in patients with a background or an increased susceptibility for the development of autoimmune diseases, clinicians should pay much attention after vaccine administration to detect and treat any adverse event quickly.

## Conclusions

In conclusion, COVID-19 has been associated with several different cutaneous manifestations, of various severity and pathophysiology, occurring both during and after SARS-CoV2 infection or vaccination. Particularly, in this systematic review we discussed all the reported cases of de novo Henoch–Schoenlein Purpura occurring after COVID-19 immunization through natural infection or vaccination. Although the precise etiopathogenetic mechanism underling this association is still unknown, we can suppose that the virus or its main antigens determines a dysregulated immune activation in susceptible patients, leading to systemic inflammation, endothelial damage and hypercoagulation state with the consequent development of vasculitis. In this scenario the hyperproduction of Immunoglobulin A may exert a leading role.

Given the evolving state of evidence we believe that, although these adverse events are overall rare, heightened awareness and timely recognition of dermatological findings in COVID-19 are important mostly in people with a personal or familiar background of immune system dysfunction. Further studies are required to better understand the pathogenetic mechanism linking HSP and COVID-19.

## Data Availability

The data that support the findings of this study are available on request from the corresponding author [M.P.].
